# When tissue is not the only issue: Poorly differentiated lung squamous-cell carcinoma with adrenal, costochondral, and cardiac metastases – case report

**DOI:** 10.3389/fonc.2023.1117024

**Published:** 2023-01-24

**Authors:** Megan Clark, Andres G. Griborio-Guzman, Nishigandha P. Burute, Sonja Lubbers, Margaret L. Anthes, Masoud Sadreddini, Olexiy I. Aseyev

**Affiliations:** ^1^ Northern Ontario School of Medicine University, Thunder Bay, ON, Canada; ^2^ Division of Cardiology, Department of Internal Medicine, Thunder Bay Regional Health Sciences Centre, Thunder Bay, ON, Canada; ^3^ Department of Diagnostic Imaging, Thunder Bay Regional Health Sciences Centre, Thunder Bay, ON, Canada; ^4^ Department of Internal Medicine, Thunder Bay Regional Health Sciences Centre, Thunder Bay, ON, Canada; ^5^ Department of Radiation Oncology, Thunder Bay Regional Health Sciences Centre, Thunder Bay, ON, Canada; ^6^ Regional Cancer Care Northwest, Thunder Bay, ON, Canada; ^7^ Department of Medical Oncology, Thunder Bay Regional Health Sciences Centre, Thunder Bay, ON, Canada

**Keywords:** squamous cell carcinoma (SCC), non-small cell lung cancer (NSCLC), cardiac imaging techniques, undifferentiated pleomorphic sarcoma (UPS), cardiac tumors, ultrasound-guided biopsy, computed tomography (CT) scan, echocardiography

## Abstract

Nonmelanoma skin cancer is the most common cancer in the world, and lung cancer is the leading cause of death from cancer. Histologically, squamous cell carcinoma (SCC) is the second most prevalent type of both skin and lung cancers. We report the case of a 38-year-old female with metastatic, poorly differentiated lung SCC detected on chest X-ray after she presented to the hospital with cough and dyspnea. She had had a 7.5 cm moderately differentiated well-circumscribed posterior scalp SCC completely excised eight years earlier. CT scan showed a large right lung mass, nodular filling defect in the left atrium (LA), and metastases to the adrenal glands and the first rib. Her pulmonary tumor extends to the LA *via* the right superior pulmonary vein, which is rarely reported in the literature. Ultrasound-guided biopsy of the rib mass showed poorly differentiated SCC. The patient received urgent radiotherapy, given superior vena cava and mainstem bronchus compression. Head CT showed no brain metastasis. A biopsy of the left adrenal initially reported an undifferentiated pleomorphic sarcoma; however, a second pathologist reported it as a poorly differentiated carcinoma of lung origin. At least three pathologists verified the specimen, and it had a PD-L1 test with a 1-49% score. An initial echocardiogram confirmed the LA mass. The patient received a Paclitaxel-Carboplatin-Pembrolizumab regimen as the first-line treatment for metastatic SCC. A repeat echocardiogram after cycle 1 showed a decrease in the size of the tumor in the LA. Almost five months after her initial visit, this young woman’s symptoms and performance status have improved post-palliative radiotherapy and chemo-immunotherapy. Follow-up CT showed smaller lung, nodal, adrenal, and costochondral masses, and evidence of necrosis. This case is clinically relevant because it represents a common problem presenting uncommonly. Moreover, it highlights that ultrasound-guided interventions and medical imaging are essential in directing metastatic cancer diagnosis, treatment, and follow-up, especially when pathology cannot confirm but only presume a specific diagnosis.

## 1 Introduction

Our report describes the case of a non-smoker 38-year-old woman with metastatic squamous cell carcinoma (SCC) of the lung and previously diagnosed with cutaneous squamous cell carcinoma (cSCC) at age 30. The patient presented to the emergency department (ED) with upper respiratory symptoms, decreased appetite and 25 kg of unintentional weight loss. She was tachycardic with a heart rate of 119 bpm and temperature of 36.3°C; otherwise, her vital signs were within normal limits on room air, and she appeared fatigued and pale. She had primary respiratory acidosis with secondary metabolic alkalosis, leukocytosis with neutrophilia, microcytic anemia, and thrombocytosis, with WBC, hemoglobin, MCV, platelets, INR, and ferritin at 21, 114, 70, 476, 1.3, and 545 µg/L respectively. She also had normal TSH, creatinine, estimated GFR, urinalysis, rheumatoid factor, ANA and anti-ENA antibodies.

Her case is unique for multiple reasons. The pathology work-up was complicated, as mentioned above and detailed in the diagnostic assessment and discussion below. For cSCC, men are most affected, with patients over 60 years old comprising 80% of nonmelanoma skin cancers (1, 2). cSCC is uncommon in patients under 50 years old, and its recurrence or metastases usually occur within five years (not eight years) in 30-50% of patients (3). On the other hand, 85-90% of lung cancer cases can be attributed to smoking (4), with adults older than 65 most affected (5, 6) and men more affected than women (7). In non-small cell lung cancer (NSCLC), only 2-3% of patients have bilateral adrenal metastases at the initial presentation (8). Even if the biopsied adrenal mass represents an undifferentiated pleomorphic sarcoma (UPS), a type of high-grade aggressive soft tissue sarcoma (STS), only 10% of patients have detectable metastases at diagnosis (9). STS primarily affects adults older than 55 years (10). The patient’s cardiac tumor extends from the right superior pulmonary vein (RSPV) into the left atrium (LA), which is rare and scarcely reported. One study examined 215 lung cancer patients with magnetic resonance angiography and found that two patients had a tumor that extended into the LA (11). A retrospective analysis of 4,668 patients who had surgery for lung cancer found evidence of pulmonary vein and LA involvement in 34 (0.7%) and 25 (0.5%) subjects (12, 13). Thus, our patient represents a minority of patients for any of these diseases. To our knowledge, this specific presentation has not been reported in the literature.

## 2 Case description

A non-smoker 38-year-old woman presented to the ED with upper respiratory symptoms, decreased appetite and 25 kg of unintentional weight loss. Her past medical history was remarkable for cSCC of the posterior scalp, diagnosed in 2015. The tumor had been growing steadily for ten years. On March 2015, a head MRI showed a 7.5 x 4.8 x 7.0 cm posterior occipital midline extracalvarial mass. This moderately differentiated well-circumscribed posterior scalp cSCC was completely excised with no evidence of lymphovascular invasion. Besides previous intermittent asthma, she had no further history of medical conditions and did not use any medications. She had worked clerical jobs and, more recently, as a leather worker, which does not involve using chemicals. Her family history included a grandfather diagnosed with multiple myeloma. She was tachycardic with a heart rate of 119 bpm and temperature of 36.3°C; otherwise, her vital signs were within normal limits on room air, and she appeared fatigued and pale.

## 3 Clinical approach and timeline

**Figure 1:**
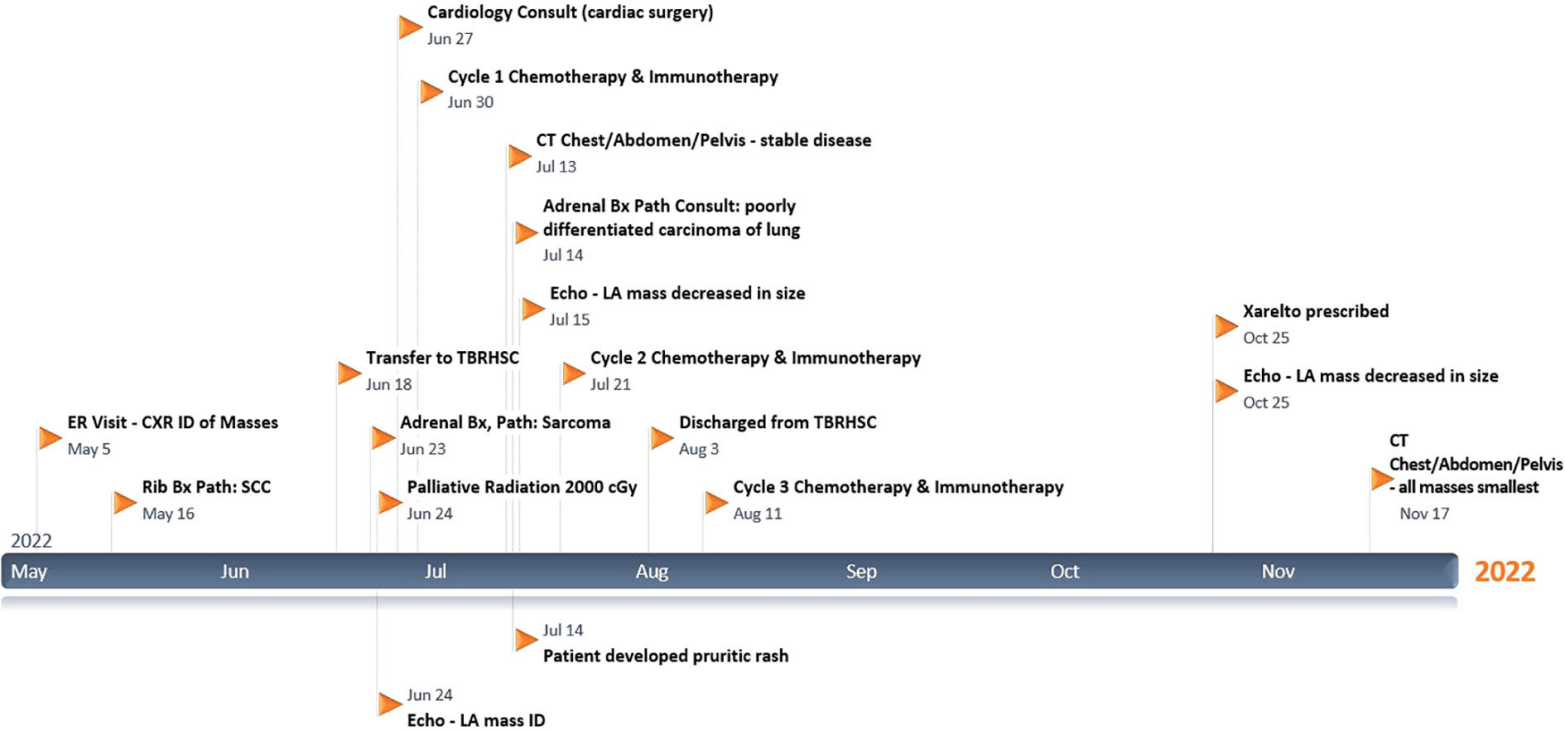
Milestone Timeline with data from the related episodes of care.

Please refer to **Figure 1** for data from the related episodes of care.

### 3.1 Diagnostic assessment

The first investigation she had on presentation to the ED was, in fact, a chest X-ray. It showed a 5 x 4.5 cm right paratracheal mass, a 14 x 11 cm mass-like opacity obscuring the right cardiac border and right lower lung (RLL), and a 3.5 x 2.8 cm subtle left upper lung (LUL) opacity projected over the first rib. The following day, she had a thoracic CT with contrast which confirmed the 11.7 x 14.1 x 13.7 cm right middle lobe (RML) and RLL mass as highly suspicious for primary lung cancer. Additionally, it showed a nodular (1.7 cm) filling defect in the LA with direct extension through the RSPV and raised the concern of a filling defect in the right atrium extending through the inferior vena cava (IVC). Finally, it also showed mediastinal and hilar lymphadenopathy, including a 4.6 x 6.3 cm right paratracheal node slightly displacing the aorta and moderately compressing the superior vena cava (SVC), thyromegaly with a left-sided nodule and metastatic lesions to the left first rib (4.9 x 3.3 cm) and both adrenal glands. That same day, a neck and abdominopelvic CT confirmed the 3.7 x 3.2 x 5.2 cm left-lower thyroid nodule requiring FNA (but “unrelated to the presentation of lung mass”) and <1 cm right-sided thyroid nodules, as well as *osteitis condensans ilii*, splenomegaly, a 2 cm hepatic and a 2.9 cm ovarian cyst, a 3.5 cm posterior wall uterine fibroid, and bilateral adrenal metastasis, measuring 8.2 x 6.0 x 3.2 cm (right) and 10.4 x 7.5 x 8.9 cm (left).

On May 13, she had ultrasound-guided biopsies of the left thyroid nodule (whose evaluation was limited by a low number of follicular cells) and the costochondral mass (which showed poorly differentiated SCC). Chest X-rays done on June 16 and June 18 showed that the multiple pulmonary masses were enlarging significantly, up to 16.4 cm (RLL), 6.8 cm (right paratracheal), and 6.2 cm (LUL). Head CT on June 19 showed no brain metastasis. An echocardiogram on June 24 estimated the baseline left ventricular ejection fraction (LVEF) at 70%, global longitudinal strain (GLS) -18%, and showed a 3.6 x 1.8 cm LA mass (from the RSPV) and an echogenic IVC (Eustachian) valve structure. Adrenal ultrasound on June 22 showed that the metastatic lesions in both glands were amenable for biopsy, hypovascular, and enlarging (12.3 x 9.1 x 6.9 cm on the right and 14.5 x 11.7 x 11.2 cm on the left). The biopsy of the left adrenal mass initially reported an UPS; however, a second pathologist reported it as a poorly differentiated carcinoma of lung origin, given the clinical history. Of note, at least three pathologists verified the specimens, including after being referred to a centre with an available pathology laboratory specializing in lung cancer. Diagnostic challenges included the adrenal biopsy being extensively necrotic and having little viable tissue available for examination and special stains. Still, immunohistochemistry was done in the biopsied specimens. The costochondral tumor was positive for P63 and negative for TTF-1 and PAX 8, excluding a primary adenocarcinoma and suggesting an SCC. It had a PD-L1 test with a 1-49% score, which guided management with Pembrolizumab as described below. The adrenal mass biopsy was negative for keratin, TTF-1, S100 protein, SOX10, smooth muscle actin (SMA), desmin, MDM2 and CDK4, and only vimentin was expressed. Occasionally SCCs can dedifferentiate and appear as spindle cell neoplasm. Multiple extrathoracic metastases make NSCLC stage IVB, and distant metastasis makes cSCC stage IV; however, studies in which most patients are like the one reported here and without previous systemic therapy are lacking to quantify her prognosis confidently.

### 3.2 Therapeutic intervention

On her first presentation, she was prescribed up to two doses of inhaled Salbutamol every 4 hours as needed. On the day of her first CT, she was started on Dexamethasone 4 mg PO to help reduce peritumoral edema. Before being transferred to Thunder Bay, she was started on Ceftriaxone 1 gr IV every 12 hours for a couple of days in case of an underlying infection. However, her leukocytosis was due to the dexamethasone use and extensive tumor burden. The Dexamethasone dose peak was 10 mg PO every 8 hours for three days during the first week of July, and by July 14, it was 4 mg PO twice a day up until discharge on August 2 with a plan to taper it over the next month. During this admission, she was also started on a Histamine-H_2_-receptor antagonist (Famotidine 20 mg PO twice a day) while on steroids and Dextromethorphan 15 mg PO every 4 hours (antitussive) as needed.

In Thunder Bay, she had her urgent palliative radiotherapy to her mediastinum and right-hemithorax (2000 cGy in 5 fractions from June 20 until June 24), given her SVC and mainstem bronchus compression by the massive lung tumor. After it was completed, a multidisciplinary discussion involving oncology, cardiology and cardiac surgery determined that, based on the patient’s performance status and disease extension, she would not tolerate cardiac surgery or a bronchoscopy, with very low chances of surviving without aggressive systemic therapy. The patient is aware of her limited second-line options if the chemo-immunotherapy fails.

Her systemic treatment was to be repeated every 21 days for four cycles. Each cycle included Pembrolizumab 2 mg/kg IV, a 50% dose-reduction of Paclitaxel (87.5 mg/m^2^ IV) and kept the dose of Carboplatin down (380-400 mg IV) to minimize possible chemo-related side effects while providing the best possible treatment option to the patient. Due to personal and logistical challenges, her fourth cycle was 29 days instead of 21 days after the previous one. Three weeks after her fourth chemo-immunotherapy cycle, she started maintenance immunotherapy with Pembrolizumab 3.65 mg/kg IV on September 29. It will be repeated every 42 days until disease progression or unacceptable toxicity up to thirteen additional doses, whichever occurs first.

### 3.3 Follow-up and outcomes

**Figure 2:**
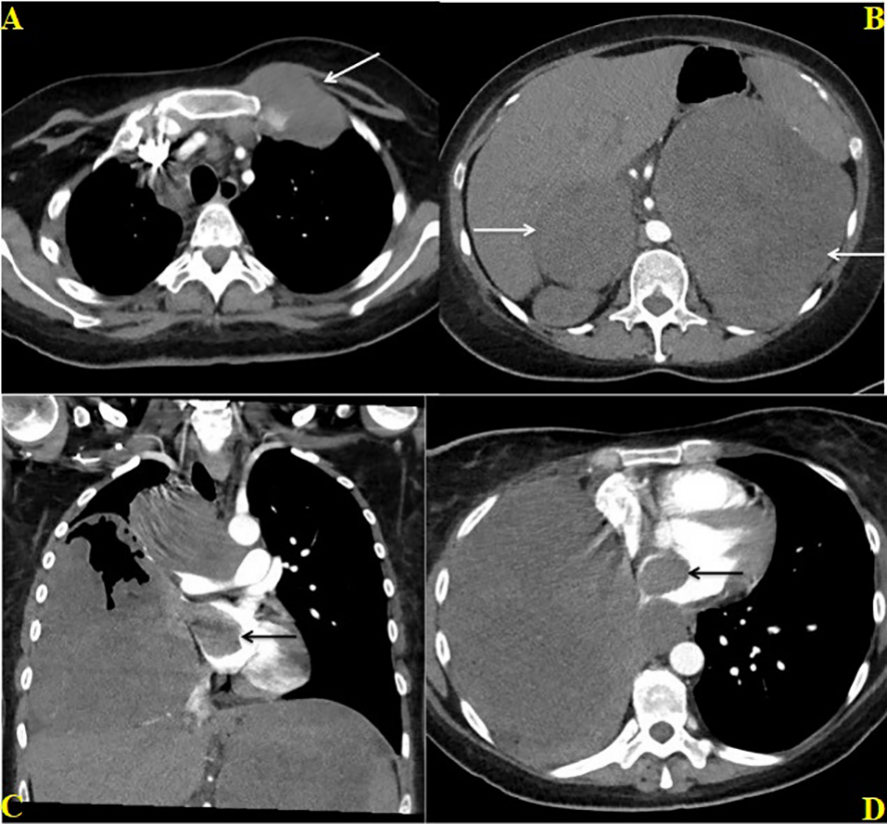
Contrast enhanced CT chest and abdomen dated June 27, 2022; Axial CT image through the chest **(A)** shows enlarged metastatic pre and paratracheal lymph nodes and a lobulated metastatic mass involving the left first rib (white arrow) at the costochondral junction. Axial CT image through the upper abdomen **(B)** shows large bilateral adrenal masses (white arrows). Coronal **(C)** and Axial **(D)** CT images through the chest shows a large hetereogenously enhancing mass in the right lung directly extending through the right superior pulmonary vein into the right atrium (black arrow) and conglomerate paratracheal lymphadenopathy **(C)**.

**Figure 3:**
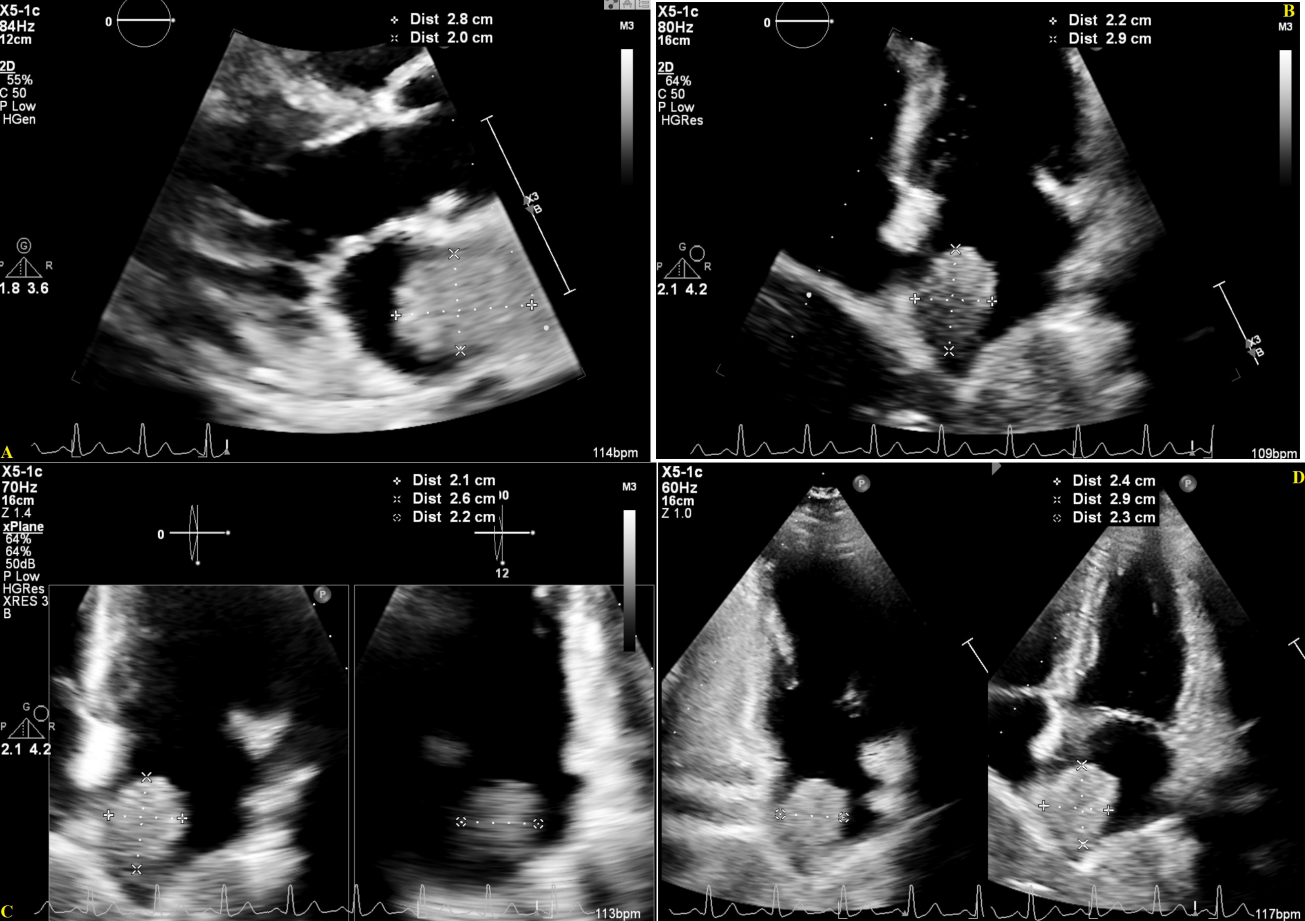
Transthoracic Echocardiogram showing a large echogenic mass in the Left Atrium, in the parasternal long-axis **(A)** and apical multi-chamber views **(B–D)**, July 26, 2022, with measures estimating a mass size of 2.6-2.9 cm x 2.0-2.4 cm.

Almost five months after her initial visit, her symptoms have significantly improved post-palliative radiotherapy and chemo-immunotherapy. Her Eastern Cooperative Oncology Group (ECOG) performance status has improved from grade 3 to 1. Follow-up diagnostic imaging includes post-radiotherapy thoracic CT in June and chest-abdominopelvic CT scans (**Figure 2**) in July and September 18, which show interesting results. Scans in June and July were consistent with continuously enlarging masses, as already mentioned, whose dimensions increased as follows: RML mass, 10 x 15 x 15-16.4 cm (including partial necrosis with vacuum phenomenon); right paratracheal node, 6.3 x 7.9 cm; left first rib, 6.4-7 x 5.7 cm; and bilateral adrenal metastasis (with some interval necrosis), measuring 10.7 x 9.8 x 9 cm (right) and 17.1 x 14.6 x 15.8 cm (left). Fortunately, CT on September 18 showed smaller lung (RML, 10 x 12.7 x 13.4 cm), nodal (right paratracheal, 5.5 x 5.6 cm), costochondral (left rib, 3.9 x 4 cm), and adrenal masses (right, 7.8 x 4.2 x 4 cm, and left, 18 x 12.6 x 12.2 cm), and increased necrosis. This CT scan also questioned a nonocclusive thrombosis in the IVC. A couple of repeat echocardiograms (**Figure 3**) in July (after chemo-immunotherapy cycles 1 and 2) confirmed the known LVEF, IVC echogenic structure and LA mass (2.9 x 2.3 cm).

Overall, she tolerated the therapeutic interventions above relatively well with an Edmonton Symptom Assessment System (ESAS) score <20, with maximum individual scores of 8 for tiredness and shortness of breath, which all improved concurrently with her ECOG performance status. On June 17, before starting radiotherapy, she developed a paraneoplastic leukemoid reaction with WBC peaking at 67.3 x 10^9^/L. The nadir of her anemia occurred one week after her first cycle of chemotherapy, with no overt hemorrhage or hemolysis and hemoglobin at 67 (for which she was transfused packed red blood cells). Her leukocytosis and anemia were resolved by September 26, with WBC and hemoglobin at 6.7 and 116, respectively. Unanticipatedly, she had developed a pruritic maculopapular rash on July 13 with unclear etiology, covering her face, especially lateral cheeks and forehead, neck, chest, upper arms, and groin. It was severe for at least a week, with thigh excoriations, arms plaques with minimal scaliness, and thoracic erythema; however, it subsided gradually with supportive care only. A dermatologist considered it a possible drug reaction likely secondary to Dextromethorphan more than Famotidine but did not think chemo-immunotherapy played a role in her eruptions.

Finally, her last echocardiogram (done on October 25) showed an LVEF >70%, a mildly reduced GLS at -18.7%, and the IVC content was again characterized as a thrombus. A recent CT scan in November and the abovementioned echocardiogram confirmed that the LA mass had reduced in size (2.5 x 1.9 cm). However, she has had palpitations, cough, and chest pain since this last month, feeling weaker. After discussing the echocardiogram findings with a Cardiologist, the Internal Medicine team prescribed her Rivaroxaban. Her November CT scan showed a large new right-sided pleural effusion and patchy consolidations deemed possibly due to post-radiation changes. Fortunately, it showed overall interval improvement. The stable IVC thrombus and the lung (RML, 9.5 x 11.3 x 12.5 cm), nodal (right paratracheal, 4.7 x 5 cm), and adrenal masses (right, 5.1 x 3 cm, and left, 10.3 x 9 cm) appeared the smallest they have been.

## 4 Discussion

A strength of this report is that the case represents a frequent problem presenting uncommonly. A multidisciplinary approach, considering clinical presentation, medical imaging, and response to therapy (as assessed clinically and by imaging tests), can overcome diagnostic and therapeutic uncertainties that can occur with challenging pathology. Our approach’s main limitations are those associated with pathology investigations. The patient presented like a textbook lung malignancy, with a sizeable pulmonary mass and metastases to the adrenals and bone. However, upon pathological inspection, this was more complex. Ultrasound-guided biopsy of the rib mass showed poorly differentiated SCC, and the left adrenal one was first considered a UPS, then a poorly differentiated carcinoma of lung origin. The uncertainty of this malignancy might be remedied with a lung biopsy. However, as Cancer Care Ontario suggests, if the patient has stage IV lung cancer, the least invasive and most accessible approach should be followed, which explains why only the rib and adrenal masses were biopsied (14).

**Figure 4:**
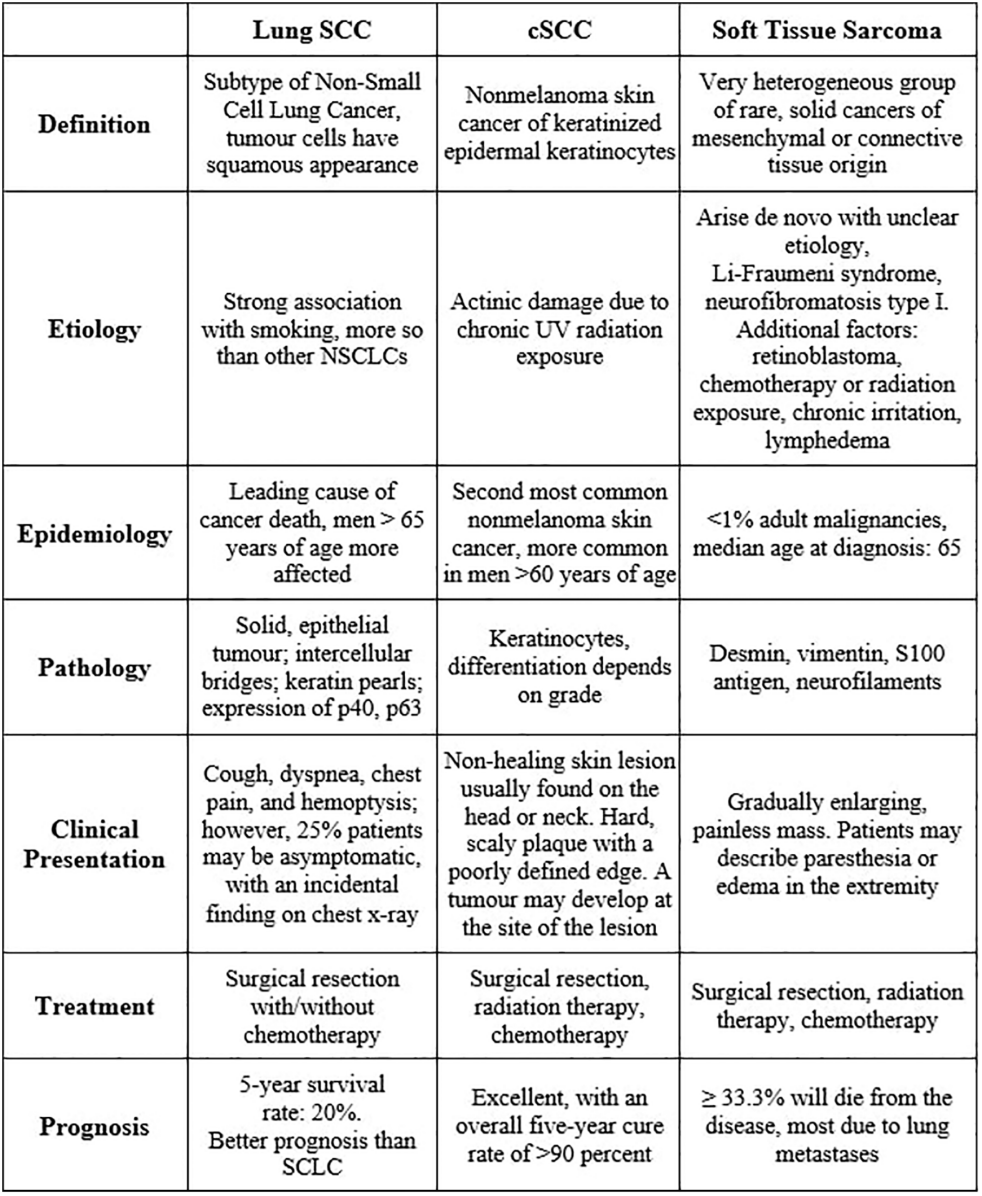
Comparison of SCC of the lung, cutaneous SCC, and soft tissue sarcoma.

One explanation for these differing diagnoses is that this patient has multiple primary malignancies. The first is the SCC which could be skin or lung, and a UPS (**Figure 4**). The lung tumor was not biopsied, so it is unclear whether there would be a third primary malignancy. The frequency of multiple primaries ranges from 2-17%. Cancer survivors may develop second primary malignancies due to an array of factors, including cancer predisposition syndromes or unique tumor characteristics, environmental exposures, and late effects of therapies (15, 16).

This patient may have a cancer predisposition syndrome, namely Li-Fraumeni syndrome. Li-Fraumeni syndrome is an inherited autosomal dominant disorder manifested by a wide range of malignancies that appear at an unusually early age (17). Li-Fraumeni syndrome is also known as Sarcoma, Breast, Leukemia, and Adrenal Gland cancer syndrome. This cancer predisposition syndrome is inherited as an autosomal dominant disorder and is associated with abnormalities in the tumor protein p53 gene (p53). Almost all types of soft tissue and bone sarcomas are seen in families with Li-Fraumeni syndrome. Patients with an abnormality in p53 who develop cancer are at a significantly increased risk of developing a second malignancy (18). For women, the lifetime risk of cancer approaches 100% and has been estimated to be ~90% by age 60 (19).

These differences in pathology can also be explained by tumor heterogeneity. Tumors can differ within a tumor (intra-tumoral heterogeneity) and between tumors (inter-tumoral heterogeneity). With intra-tumoral heterogeneity, histological examination often shows notable differences in the morphology of cancer cells in different areas of the same lesion, and areas of necrosis may be present. Also, the cells in a tumor may be cycling or non-cycling, quiescent, or reproductively dead (20). The stage of the cell cycle can influence cellular properties such as membrane biochemistry (21) and the ability to metastasize (22). Also, growth patterns, differentiation and keratinization grades between the primary and metastatic tumors can differ (23).

Lastly, it is also essential to consider the neglect of the first malignancy. The patient neglected the first cSCC for ten years and did not share with us why she waited so long to seek medical attention. Denial is a coping mechanism to adapt to stressful situations (24–26). It can become maladaptive when it leads to behaviors that are detrimental to their care, e.g., delaying seeking treatment. Many psychological, physiological, and demographic factors are present in those who show maladaptive denial (27), such as poor medical literacy, socioeconomic stresses, and tumors that grow slowly, as in our patient’s case (28). Neglecting this high-risk malignancy might have had a role in cancer recurrence, categorized as such due to its size and invasion of the aponeurosis. Since no studies have been conducted with similar populations, the prognosis for the patient is unclear, especially if immunotherapy does not succeed. The patient would have limited options beyond a palliative approach in this case.

Clinical presentation and pathology can be quite different. Despite the pathology challenges, the patient responded well to the Paclitaxel-Carboplatin-Pembrolizumab regimen as the first-line treatment for metastatic SCC. The patient was treated accordingly based on clinical presentation and a multidisciplinary decision. Imaging studies confirmed the stabilization and improvement of her disease, already hinted clinically with the resolution of her dyspnea and cough. This treatment resulted in stable disease and lessened symptom burden. Tumors may be heterogenous, and immunohistochemistry markers are not 100% sensitive. Thus, medical imaging is essential in directing metastatic cancer diagnosis, treatment, and follow-up, especially when pathology cannot confirm but only presume a specific diagnosis.

## Data availability statement

The original contributions presented in the study are included in the article/supplementary material. Further inquiries can be directed to the corresponding author.

## Ethics statement

Written informed consent was obtained from the individual(s) for the publication of any potentially identifiable images or data included in this article.

## Author contributions

All the listed authors contributed substantially to the conception or design of the work or the acquisition, analysis, or interpretation of data for the work. They drafted the work or revised it critically for important intellectual content and provided approval for publication of the content. All authors agree to be accountable for all aspects of the work in ensuring that questions related to the accuracy or integrity of any part of the work are appropriately investigated and resolved. All authors contributed to the article and approved the submitted version.

## References

[1] Firnhaber JM . Diagnosis and treatment of basal cell and squamous cell carcinoma. Am Fam Physician (2012) 86:161–8. 22962928

[2] Diffey BL Langtry JAA . Skin cancer incidence and the ageing population. Br J Dermatol (2005) 153:679–80. doi: 10.1111/J.1365-2133.2005.06799.X 16120172

[3] Schmults CD Blitzblau R Aasi SZ Alam M Andersen JS Baumann BC . NCCN guidelines version 2.2022 squamous cell skin cancer (2022). Available at: https://www.nccn.org/home/member-.

[4] Ettinger DS Wood DE Aisner DL Akerley W Bauman JR Bharat A . NCCN guidelines version 5.2022 non-small cell lung cancer continue NCCN guidelines panel disclosures (2022). Available at: https://www.nccn.org/home/member-.

[5] Planchard D Popat S Kerr K Novello S Smit EF Faivre-Finn C . Metastatic non-small cell lung cancer: ESMO clinical practice guidelines for diagnosis, treatment and follow-up. Ann Oncol (2018) 29:iv192–237. doi: 10.1093/annonc/mdy275 30285222

[6] Novello S Barlesi F Califano R Cufer T Ekman S Levra MG . Metastatic non-small-cell lung cancer: ESMO clinical practice guidelines for diagnosis, treatment and follow-up. Ann Oncol (2016) 27. doi: 10.1093/annonc/mdw326 27664245

[7] Siegel RL Miller KD Fuchs HE Jemal A . Cancer statistics, 2022. CA Cancer J Clin (2022) 72:7–33. doi: 10.3322/caac.21708 35020204

[8] Karanikiotis C Tentes AA Markakidis S Vafiadis K . Large Bilateral adrenal metastases in non-small cell lung cancer. World J Surg Oncol (2004) 2:1–7. doi: 10.1186/1477-7819-2-37/TABLES/1 15541184 PMC535544

[9] WHO Classification of Tumours Editorial Board . Soft tissue and bone tumours. 5th ed. (Lyon, France: International Agency for Research on Cancer Press) (2020).

[10] Nystrom LM Reimer NB Reith JD Dang L Zlotecki RA Scarborough MT . Multidisciplinary management of soft tissue sarcoma. Sci World J (2013) 2013. doi: 10.1155/2013/852462 PMC3745982 23983648

[11] Takahashi K Furuse M Hanaoka H Yamada T Mineta M Ono H . Pulmonary vein and left atrial invasion by lung cancer: Assessment by breath-hold gadolinium-enhanced three-dimensional MR angiography. J Comput Assist Tomogr (2000) 24:557–61. doi: 10.1097/00004728-200007000-00008 10966186

[12] Stella F Dell’Amore A Caroli G Dolci G Cassanelli N Luciano G . Surgical results and long-term follow-up of T4-non-small cell lung cancer invading the left atrium or the intrapericardial base of the pulmonary veins. Interact Cardiovasc Thorac Surg (2012) 14:415–9. doi: 10.1093/icvts/ivr160 PMC3309831 22269143

[13] Riquet M Grand B Arame A Pricopi CF Foucault C Dujon A . Lung cancer invading the pericardium: Quantum of lymph nodes. Ann Thorac Surg (2010) 90:1773–7. doi: 10.1016/J.ATHORACSUR.2010.07.039 21095307

[14] Cancer Care Ontario . Lung cancer imaging guidelines: Integration with the lung cancer diagnosis and staging clinical pathway (2014). Available at: https://www.cancercareontario.ca/en/guidelines-advice/types-of-cancer/3201.

[15] Coyte A Morrison DS McLoone P . Second primary cancer risk - the impact of applying different definitions of multiple primaries: Results from a retrospective population-based cancer registry study. BMC Cancer (2014) 14:1–11. doi: 10.1186/1471-2407-14-272 24742063 PMC4005906

[16] Vogt A Schmid S Heinimann K Frick H Herrmann C Cerny T . Multiple primary tumours: Challenges and approaches, a review. ESMO Open (2017) 2:e000172. doi: 10.1136/esmoopen-2017-000172 28761745 PMC5519797

[17] Malkin D . Li-Fraumeni syndrome. Genes Cancer (2011) 2:475–84. doi: 10.1177/1947601911413466 PMC3135649 21779515

[18] Bougeard G Renaux-Petel M Flaman JM Charbonnier C Fermey P Belotti M . Revisiting Li-fraumeni syndrome from TP53 mutation carriers. J Clin Oncol (2015) 33:2345–52. doi: 10.1200/JCO.2014.59.5728 26014290

[19] Mai PL Best AF Peters JA DeCastro RM Khincha PP Loud JT . Risks of first and subsequent cancers among TP53 mutation carriers in the national cancer institute Li-fraumeni syndrome cohort. Cancer (2016) 122:3673–81. doi: 10.1002/cncr.30248 PMC5115949 27496084

[20] Heppner GH Miller BE . Tumor heterogeneity: biological implications and therapeutic consequences. Cancer Metastasis Rev (1983) 2:5–23. doi: 10.1007/BF00046903 6616442

[21] Pasternak CA Warmsley AMH Thomas DB . STRUCTURAL ALTERATIONS IN THE SURFACE MEMBRANE DURING THE CELL CYCLE. J Cell Biol (1971) 50:562. doi: 10.1083/jcb.50.2.562 4107022 PMC2108270

[22] Suzuki N Withers HR Koehler MW . Heterogeneity and variability of artificial lung colony-forming ability among clones from mouse fibrosarcoma. Cancer Res (1978) 38:3349–51. 688224

[23] Ichinose J Shinozaki-Ushiku A Takai D Fukayama M Nakajima J . Differential diagnosis between primary lung squamous cell carcinoma and pulmonary metastasis of head and neck squamous cell carcinoma. Expert Rev Anticancer Ther (2016) 16:403–10. doi: 10.1586/14737140.2016.1147352 26813704

[24] Siemerink EJM Jaspers JPC Plukker JTM Mulder NH Hospers GAP . Retrospective denial as a coping method. J Clin Psychol Med Settings (2011) 18:65–9. doi: 10.1007/S10880-011-9223-X PMC3058364 21359504

[25] Hackett TP Cassem NH . Development of a quantitative rating scale to assess denial. J Psychosom Res (1974) 18:93–100. doi: 10.1016/0022-3999(74)90072-5 4436841

[26] Vos MS de Haes JCJM . Denial in cancer patients, an explorative review. Psychooncology (2007) 16:12–25. doi: 10.1002/PON.1051 16868929

[27] Alam M Goldberg LH Silapunt S Gardner ES Strom SS Rademaker AW . Delayed treatment and continued growth of nonmelanoma skin cancer. J Am Acad Dermatol (2011) 64:839–48. doi: 10.1016/j.jaad.2010.06.028 21055843

[28] Varga E Korom I Raskó Z Kis E Varga J Oláh J . Neglected basal cell carcinomas in the 21st century. J Skin Cancer (2011) 2011. doi: 10.1155/2011/392151 PMC2993023 21151693

